# Pinhole small-angle neutron scattering based approach for desmearing slit ultra-small-angle neutron scattering data

**DOI:** 10.1107/S1600576724008380

**Published:** 2024-09-25

**Authors:** Vasyl Ryukhtin, Adél Len, László Almásy, Ewa Juszyńska-Gałązka, Wojciech Zając, Oleksandr Tomchuk

**Affiliations:** a Neutron Physics Laboratory Nuclear Physics Institute ASCR 250 68Řež Czechia; b Neutron Spectroscopy Department, HUN-REN Centre for Energy Research, 1121Budapest, Hungary; c Faculty of Engineering and Information Technology University of Pécs 7624Pécs Hungary; d Department of Soft Matter Research The Henryk Niewodniczański Institute of Nuclear Physics, Polish Academy of Sciences 31-342Kraków Poland; e ISIS Neutron and Muon Source Rutherford Appleton Laboratory DidcotOX11 0QX United Kingdom; Universität Duisburg-Essen, Germany

**Keywords:** small-angle neutron scattering, SANS, ultra-small-angle neutron scattering, USANS, desmearing, slit geometry

## Abstract

Presented here is an effective approach to desmearing slit ultra-small-angle neutron scattering (USANS) data, based on complementary small-angle neutron scattering (SANS) measurements, leading to a seamless merging of these data sets. The study focuses on the methodological aspects of desmearing USANS data, which can then be presented in the conventional manner of SANS, enabling a broader pool of data analysis methods. The key innovation lies in the use of smeared SANS data for extrapolating slit USANS, offering a self-consistent integrand function for desmearing with Lake’s iterative method. The proposed approach is validated through experimental data on porous anodized aluminium oxide membranes, showcasing its applicability and benefits. The findings emphasize the importance of accurate desmearing for merging USANS and SANS data in the crossover *q* region, which is particularly crucial for complex scattering patterns.

## Abstract 2

This study presents a new, methodologically robust approach for accurately desmearing slit-smeared ultra-small-angle neutron scattering (USANS) data. The proposed technique, validated on porous anodized aluminium oxide membranes, facilitates the precise merging of USANS and small-angle neutron scattering data, offering improved data analysis capabilities in the mesoscale structural research domain.

## 1. Introduction

For several decades, small-angle neutron scattering (SANS) in pinhole geometry has been strengthening its position as a tool for analysing various mesoscale structures (Brumberger, 1995 ▸; Jeffries *et al.*, 2021 ▸). The analysis of the morphology and composition of scattering objects often provides unique information not accessible to X-ray, electron or atomic force methods, particularly concerning light or magnetic chemical elements and various buried structures. On the nanometre scale, small-angle scattering finds widespread applications across a variety of research subjects, including biological ones (Jeffries *et al.*, 2021 ▸; Mühlbauer *et al.*, 2019 ▸; Krueger, 2022 ▸; Liu *et al.*, 2022 ▸).

The limitations of the method’s capabilities concerning the submicrometre size scale are primarily determined by the instrumental resolution defined by the minimum wavevector transfer *q*_min_ [*q* = (4π/λ) sin θ, where θ is half the scattering angle and λ is the wavelength of the incident radiation]. Expansions of the *q* range towards low values are implemented either by increasing the sample-to-detector path (Lindner *et al.*, 1992 ▸; Wood *et al.*, 2018 ▸) or by decreasing the transmitted beam size on the detector, allowing the use of a smaller beamstop. This is usually complemented by using a higher-resolution detector and focusing neutron optics (Pipich & Fu, 2015 ▸; Boukheir *et al.*, 2017 ▸; Iwase *et al.*, 2011 ▸; Koizumi & Noda, 2019 ▸; Galvan Josa *et al.*, 2022 ▸). However, a decrease in *q*_min_ by orders of magnitude (ultra-small-angle neutron scattering, USANS) can only be achieved with high-resolution double-crystal diffractometers of the Bonse–Hart type (Bonse & Hart, 1966 ▸; Carpenter & Agamalian, 2010 ▸). As of today, these instruments include Kookaburra at ANSTO (Australia) (Rehm *et al.*, 2018 ▸), BT5 USANS at NIST (USA) (Barker *et al.*, 2005 ▸), BL-1A USANS at SNS ORNL (USA) (Agamalian *et al.*, 2018 ▸), S18 USANS at ILL (France) (Kroupa *et al.*, 2000 ▸), DCD at JRR-3 (Japan) (Aizawa & Tomimitsu, 1995 ▸), USANS at TU Wien (Austria) (Jericha *et al.*, 2007 ▸), the ECHO setup on MORPHEUS at PSI (Switzerland), KIST-USANS at HANARO (South Korea) (Kim *et al.*, 2016 ▸), USANS at CMRR (China) (Peng *et al.*, 2022 ▸) and MAUD at NPI ASCR (Czechia) (Strunz *et al.*, 1997 ▸). These facilities are extensively used in areas of structural research (Schaefer & Agamalian, 2004 ▸; Bhatia, 2005 ▸; Weir *et al.*, 2016 ▸; Rehm *et al.*, 2013 ▸; Nolan *et al.*, 2017 ▸; Tomchuk *et al.*, 2020 ▸, 2021 ▸; Ryukhtin *et al.*, 2023 ▸).

**Figure 1:**
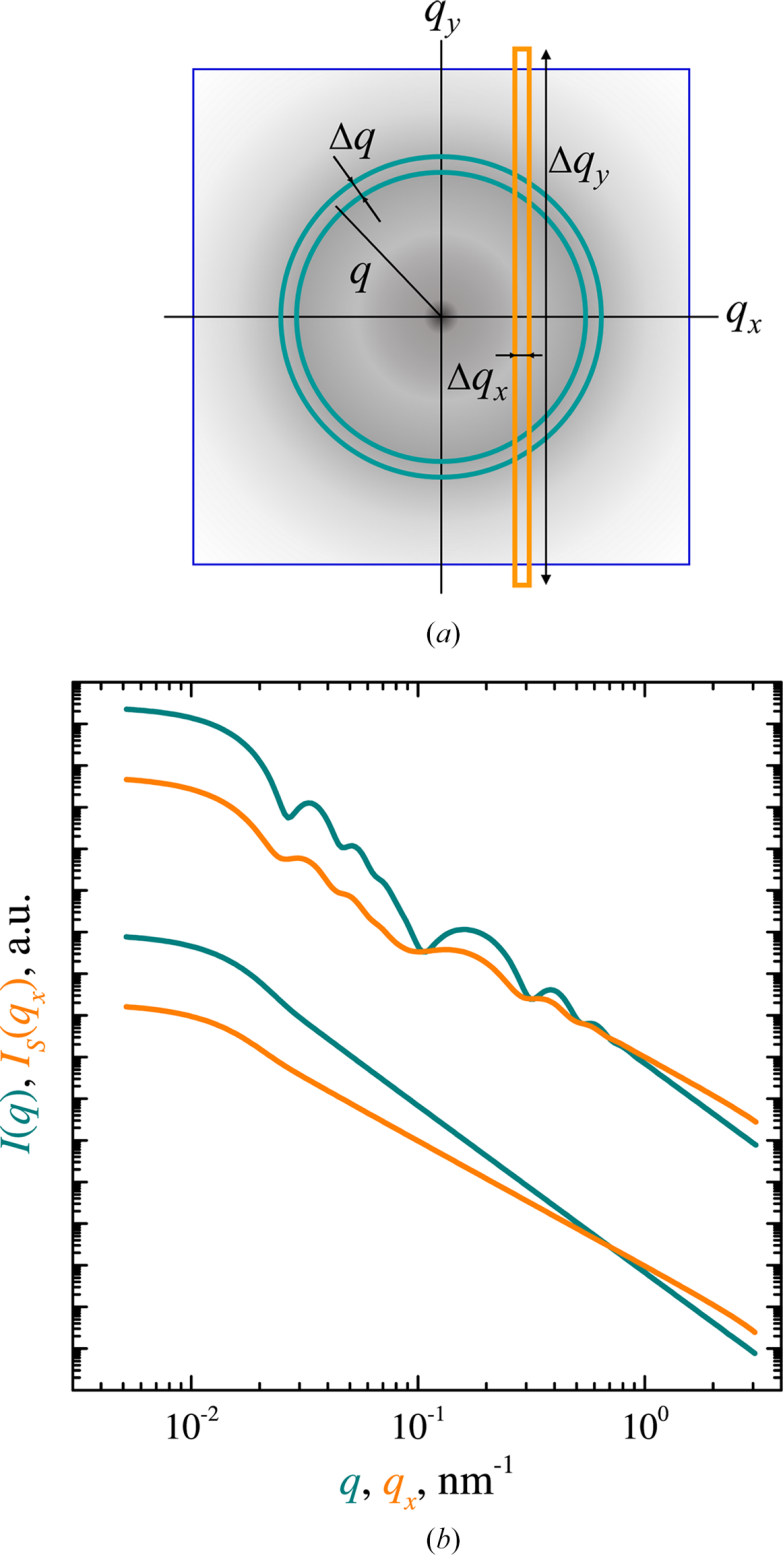
(*a*) Two types of grouping of 2D small-angle scattering data in *q* space: azimuthal averaging for pinhole geometry (turquoise annulus) and linear averaging for slit geometry (orange rectangle). (*b*) Examples of pinhole (turquoise) and slit (orange) small-angle scattering patterns for two types of objects: (top) low-polydispersity core–shell particles and (bottom) highly polydisperse homogeneous spheres. Detailed structural parameters are given in Section 3.

The USANS technique is based on a double-crystal geometry, which provides high resolution in the horizontal plane but much lower resolution in the vertical plane. The basic difference between USANS and conventional SANS instruments lies in the method of data collection in the detector plane [Fig. 1 ▸(*a*)]. This leads to a significant gain in signal intensity at low transmitted vectors, but at the cost of additional smoothing (oversmoothing, smearing) of the scattering curves [Fig. 1 ▸(*b*)]. Due to such (strong) distortion, the slit-smeared data cannot be directly and seamlessly augmented with azimuthally symmetric SANS data.

In principle, it is possible to fit both data sets simultaneously, USANS and SANS, accounting for the appropriate instrumental smearing (Šaroun, 2000 ▸, 2007 ▸; Tomchuk *et al.*, 2019 ▸). However, desmearing USANS data should be considered a more user-friendly approach, enabling the presentation of these data in the usual manner of SANS, supported by a broader range of data analysis methods. Lake’s iterative method (Lake, 1967 ▸), described in more detail below, has proved to be effective for this purpose. It facilitates obtaining a desmeared scattering curve employing trial functions, which must be integrated over a sufficiently wide *q* range extending up to the vertical instrumental resolution Δ*q*_*y*_. As the high-*q* part of this range is not accessible by a USANS instrument, the experimental data are typically extrapolated using a power-law function (Kline, 2006 ▸). While this approach yields robust results, we demonstrate in this article that, for certain non-trivial cases, the desmearing of USANS data extended with such a power-law extrapolation gives an erroneous scattering curve at large *q*, leading to incorrect merging of the USANS data with conventional pinhole SANS. Here, we suggest an intuitively comprehensible procedure to address this issue, relying on the use of artificially slit-smeared SANS data to extrapolate the experimental USANS data. This approach excludes uncertainties at the desmearing stage and provides a flawless matching of data in the overlapping USANS/SANS range. This desmearing approach was successfully tested on scattering data from aluminium oxide nanoporous membranes.

## 2. Applied USANS theory

### 2.1. Slit-smeared scattering data

In the framework of the classical setup of a pinhole SANS experiment, the isotropic differential scattering cross section per unit sample volume or the scattering intensity, *I*(*q*), is recorded as a function of the modulus of the transmitted wavevector *q*. The transition from azimuthal averaging of the two-dimensional scattering pattern to slit averaging [Fig. 1 ▸(*a*)] can be mathematically represented as an integration over one of the projections of the transmitted wavevector (Brumberger, 1995 ▸):
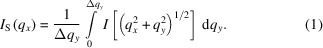
To extrapolate to the zero value of the scattering vector, the assumption *I*(*q*) = *I*(*q*_min_) for *q* < *q*_min_ can be used, since in the typical case of micrometre-sized structural inhomogeneities, the interval (0, *q*_min_) makes a negligible contribution to the integral in equation (1). Equation (1) conveys the primary concept behind employing slit collimation.

### 2.2. Lake’s iterative desmearing algorithm

Since it is not possible to solve the integral of equation (1) directly to obtain *I*(*q*) based on *I*_S_(*q*_*x*_), Lake proposed a procedure based on analysis of the *I*(*q*)/*I*_S_(*q*_*x*_ = *q*) ratio (Lake, 1967 ▸) by searching for a trial function *I*_*N*_(*q*), which converges to *I*_*N*S_(*q*) = *I*_S_(*q*) after several iterations *N* using the following equation:
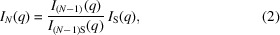
where the smearing occurs by trapezoidal integration as described by equation (1).

According to Lake, if the experimentally measured intensity of USANS is used as the zero trial function, then only *N* = 4 iterations are sufficient to achieve the coincidence *I*_4S_(*q*) ≃ *I*_S_(*q*) within the limits of experimental error. Theoretically, increasing the number of iterations should improve the result for fairly smooth functions. However, for noisy experimental data, using large values of *N* can lead to the appearance of artefacts.

### 2.3. Experimental aspects

**Figure 2:**
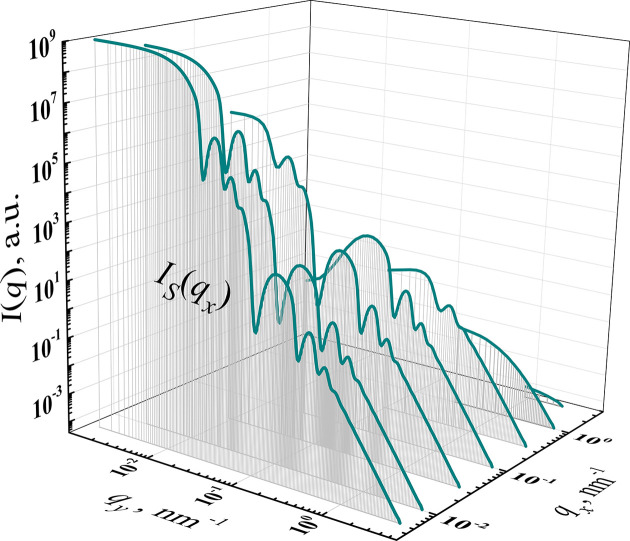
A representation of the slit-smearing procedure [equation (1)]. For each *q*_*x*_, the scattering intensity *I*(*q*) is integrated as a function of *q*_*y*_ available [equation (3)].

The only data that are available for the desmearing procedure are, of course, the array {*q*_*xi*_, *I*_S*i*_, δ*I*_S*i*_} obtained after appropriate experimental corrections related to instrumental resolution, detector pixel sensitivity *etc*. Ideally, the data should be free from instrumental smearing, but pinhole-geometry smeared data can also be used with sufficient accuracy, as shown in the practical examples below. Since the integration in equation (1) is performed over the variable *q*_*y*_, an array of real positive values {*q*_*yj*_} should be determined for each value of *q*_*xi*_ = *q*_*i*_:
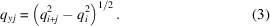
Consequently, for each *q*_*xi*_, the integrand function *I*(*q*) is different (Fig. 2 ▸).

Regarding the determination of the absolute values of the experimental errors in the intensity of the desmeared data, the best approach involves preserving the relative errors by proportionally scaling the slit experimental data:
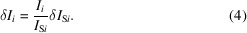


## 3. Materials and methods

The *SasView* software (Version 5.0.5; https://www.sasview.org/) was used for modelling of the scattering curves. The curves were calculated over the range 0.005–4 nm^−1^ using a logarithmic scale for *q* values. Two types of particle in a medium with zero scattering length density were considered, namely homogeneous and core–shell. In the case of homogeneous particles, these were polydisperse spheres with an average radius of 100 nm, distributed according to the normal law (PD ratio 50%). For the core–shell particle model, a core radius of 150 nm and a shell thickness of 30 nm were used. Both sizes were Gaussian distributed (PD ratio 10%). The core and shell scattering length densities were fixed in a ratio of 2:1.

The mesoporous anodized aluminium oxide (AAO) membranes used in this study were in the form of 13 mm diameter disc wafers, 50 µm thick, and were purchased from Synkera Technologies Inc. (Longmont, USA). Scanning electron microscopy data were acquired using a Phenom Pro X microscope (Phenom-World, Eindhoven, Netherlands) equipped with a CeB_6_ electron source and a four-segment backscattered electron detector. It was confirmed that the nanochannels are cylindrical. Several pore diameters were used: 20, 40 and 200 nm.

USANS experiments were performed on the double-crystal high-resolution diffractometer MAUD (NPI ASCR, Řež, Czechia). Unlike conventional double-crystal diffractometers, MAUD is equipped with an elastically bent Si crystal monochromator and analyser in fully asymmetric diffraction geometry and uses a neutron wavelength of 0.2 nm (Strunz *et al.*, 1997 ▸). The curvature level of these crystals is changeable, which allows tuning of the instrumental resolution. Combining the three instrumental resolutions covers a total *q*_*x*_ range from about 0.002 to 0.2 nm^−1^. The angular deviation of the scattered neutrons is registered by a position-sensitive detector, avoiding an angular scan of the analyser. Conventional SANS measurements were carried out using the Yellow Submarine SANS instrument (Budapest Neutron Centre, Hungary) at neutron wavelengths of 0.37 and 0.85 nm (monochromatization Δλ/λ = 20%) (Almásy, 2021 ▸). The beam diameter on the sample was 8 mm. The standard calibration procedure using 1 mm thick H_2_O was used. The instrumental parameters are summarized in Table 1 ▸.

## 4. Results and discussion

The desmearing approach described above involves the integration in equation (1) up to the upper limit Δ*q*_*y*_, which is equal to the maximum extent of vertical resolution of the instrument. For example, this parameter equals 1.17 nm^−1^ for BT5 USANS at NIST (Barker *et al.*, 2005 ▸) and about 1.0 nm^−1^ for MAUD at NPI ASCR (Strunz *et al.*, 1997 ▸). However, in practice, the maximum experimentally achievable *q*_*x*_ on slit USANS instruments is an order of magnitude smaller. In the literature, Kline (2006 ▸) proposed the use of a power-law extrapolation of the experimental data up to *q*_*x*_ = Δ*q*_*y*_ to use Lake’s algorithm [equations (1) and (2)]. This approach works well in some cases, such as highly polydisperse homogeneous particles, since the corresponding scattering curve does not contain a distinct set of extremes [Fig. 1 ▸(*b*)].

Our hypothesis was that if the power-law extrapolation does not adequately reflect the true scattering pattern, it may have a significant impact on the reconstructed *I*(*q*) function since, according to equation (3), intensities at high values of *q*_*x*_ are used for calculations at low and intermediate *q*.

**Figure 3:**
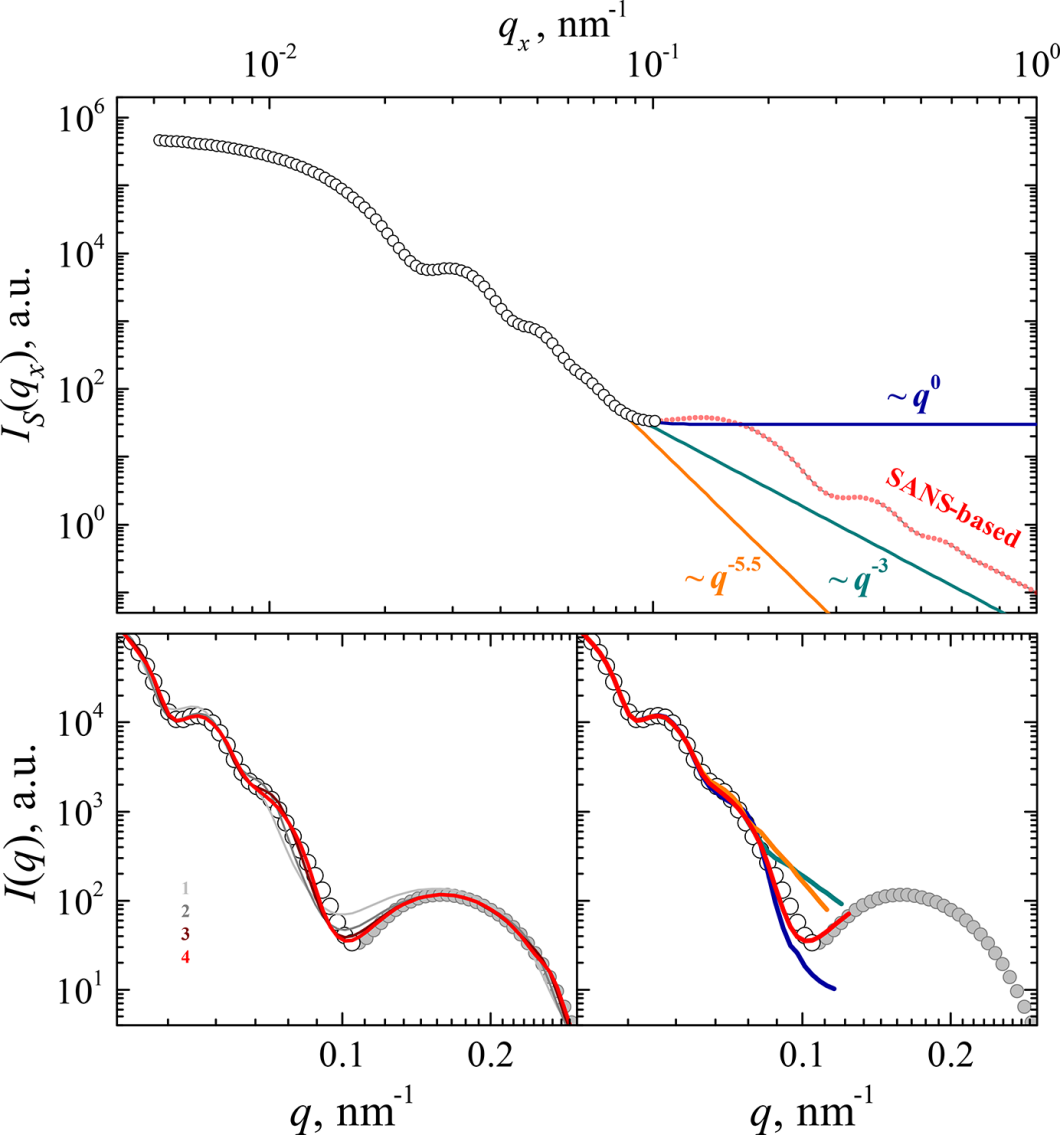
Slit USANS model data for a specific core–shell particle system, limited to *q*_max_ = 0.1 nm^−1^ (open circles), and either extrapolated by power laws or extended using smeared pinhole SANS data. The lower-right panel shows the corresponding desmeared data (lines) compared with the original pinhole data in both the USANS (white circles) and SANS (grey circles) ranges. The lower-left panel demonstrates the performance of the proposed approach for different numbers of iterations.

Fig. 3 ▸ illustrates different options for extrapolating USANS data, using the example of a scattering curve for spherical core–shell particles. The structural parameters (Section 3) were selected such that *q*_*x*max_ corresponds to a local minimum of the scattering curve at *q* = 0.1 nm^−1^ (Fig. 3 ▸). The power-law dependence in the 0.1–1 nm^−1^ range can be obtained either by fitting experimental data in the 0.05–0.1 nm^−1^ interval or by using Porod’s law, *q*^−3^. Additionally, the option of extrapolation by a constant was considered, as when the intensity is reduced to a very low level the scattering signal becomes practically flat and similar to the incoherent background. These three cases are shown, together with the new desmearing approach where the scattering intensity *I*_S_(*q*_*x*_) in the 0.1–1 nm^−1^ interval corresponds to the true slit-smeared scattering function.

As shown in the inset to Fig. 3 ▸, for all four types of extrapolation, the calculated *I*(*q*) functions coincide in the small and intermediate *q* ranges for *N* = 4 iterations. Significant differences appear only near *q*_max_ = 0.1 nm^−1^. This is a natural consequence of the fact that the contribution of the high-*q* range to the integration in equation (1) is small for *q*_*x*_ values far from *q*_*x*max_.

Nevertheless, the resulting intensities at the tails of the desmeared USANS curves differ significantly – the difference in the case shown above is one order of magnitude. This can lead to erroneous merging of the experimental desmeared USANS and original SANS curves, which can affect the interpretation of the experimental data. For more complex scattering curves, such as those with complex peak structures for highly ordered supramolecular systems, the accuracy of the extrapolation could play an even more significant role.

**Figure 4:**
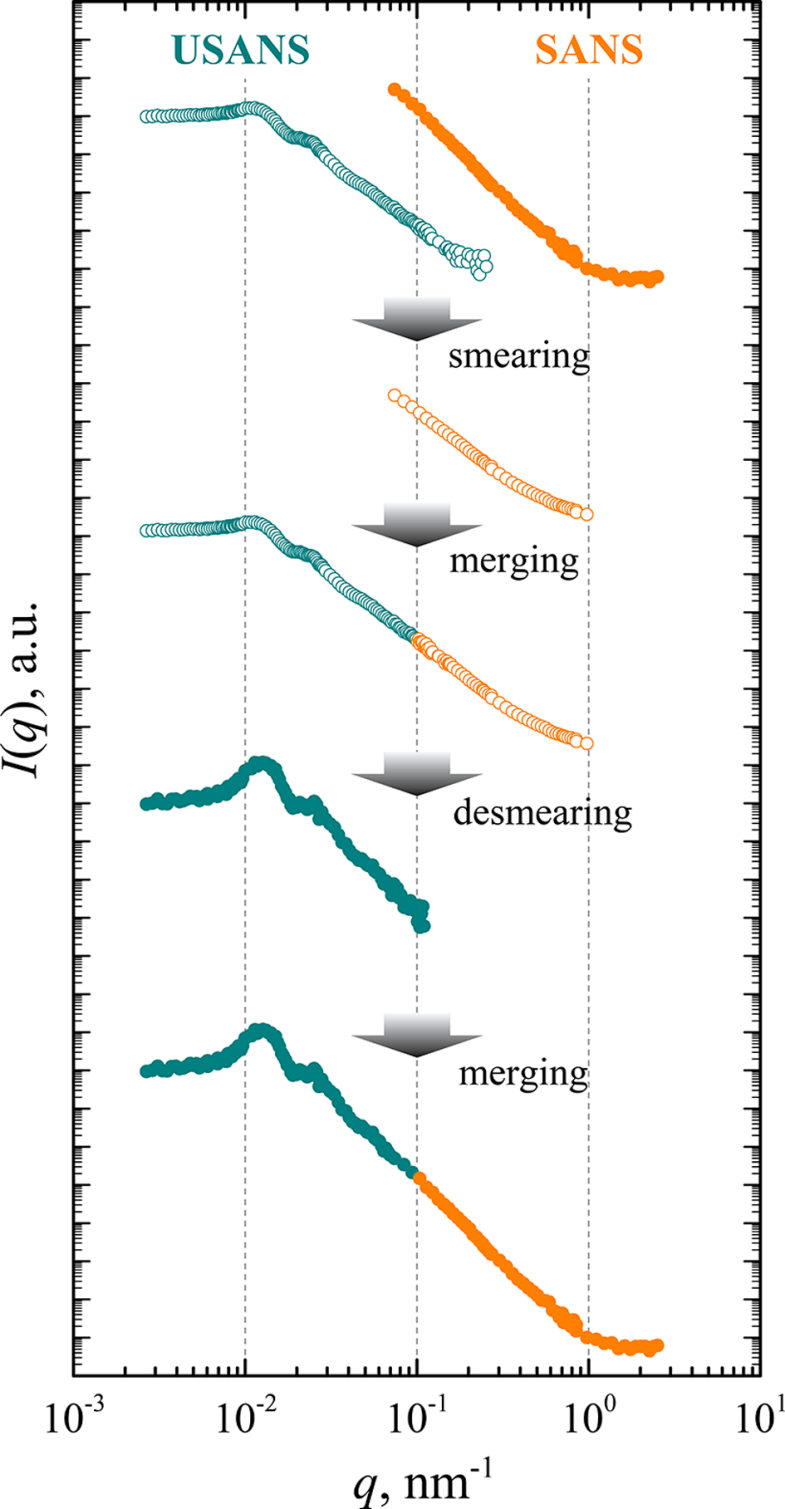
A schematic diagram of the proposed approach for desmearing USANS data. From top to bottom, (i) smearing complementary SANS data, (ii) merging USANS and SANS data in slit geometry, (iii) desmearing USANS data according to Lake’s algorithm, and (iv) merging USANS and SANS data in pinhole geometry. Filled circles are the scattering intensity in the pinhole representation, while empty circles are intensity in the slit representation for the AAO membrane sample with 200 nm cylindrical pores.

We propose a simple and straightforward approach, summarized in Fig. 4 ▸. We suggest the use of slit-smeared experimental pinhole SANS data for the extrapolation of USANS data used in the first step of the desmearing procedure. This way an experimentally substantiated and self-consistent integrand function for Lake’s algorithm in desmearing the USANS data is obtained. The correctly desmeared USANS data can then be matched and merged with the original SANS data in the crossover region, particularly in the vicinity of 0.1 nm^−1^.

The proposed approach was successfully used in the treatment of neutron scattering data on porous AAO membranes. This material is finding more and more interesting applications; for example, it can provide spatial confinement for manipulating the physicochemical properties of liquid-crystalline phases of low molecular weight organic compounds (Jasiurkowska-Delaporte *et al.*, 2021 ▸; Juszyńska-Gałązka & Zając, 2023 ▸).

**Figure 5:**
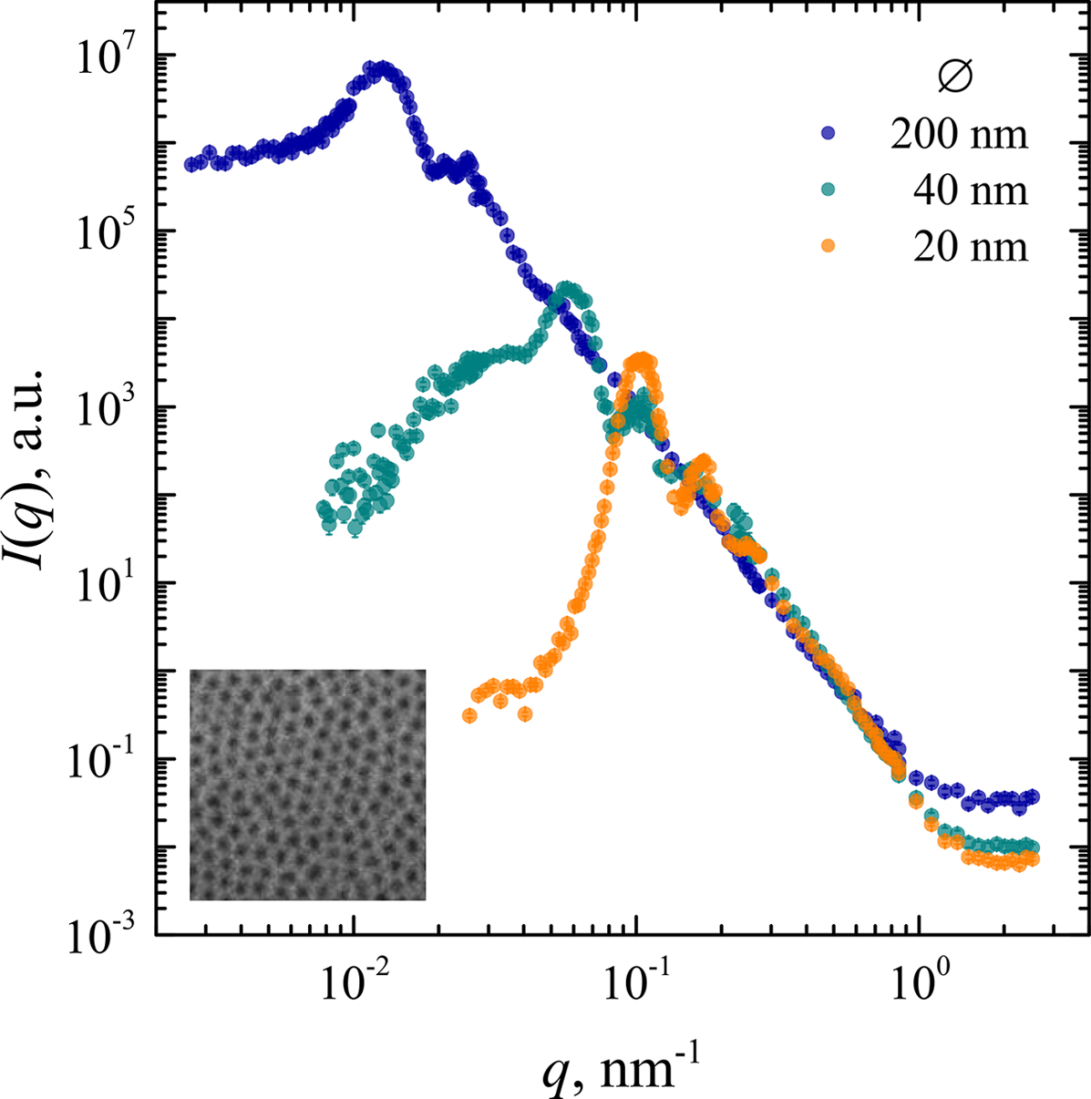
The desmeared experimental data obtained using the proposed approach by desmearing USANS data from the MAUD instrument using SANS data from the Yellow Submarine instrument. The samples are AAO membranes with cylindrical pores of nominal diameters 20, 40 and 200 nm. A scanning electron microscopy image of the AAO sample with 20 nm diameter pores is shown in the inset.

The SANS experiment in pinhole geometry was conducted on the Yellow Submarine instrument at the Budapest Neutron Centre, and that in the slit geometry on the MAUD instrument of the Nuclear Physics Institute ASCR (instrument details are summarized in Section 3). The resulting scattering curves for samples with various diameters of cylindrical pores are presented in Fig. 5 ▸. Using the proposed approach, it was possible to cover a wide range of transmitted wavevectors *q*, which in the case of an AAO membrane with pores of 200 nm diameter reached three orders of magnitude in *q*.

Note that the presented approach is entirely applicable when working with small-angle and ultra-small-angle X-ray scattering data (Ilavsky *et al.*, 2009 ▸).

## 5. Conclusions

The modern demands of the SANS user community require expanding the *q* interval and have led to the active use of double-crystal diffractometers to cover the ultra-small-angle range. In this work, the use of conventional pinhole SANS data for desmearing slit-smeared USANS data is proposed and justified. This approach allows the avoidance of the uncertainties caused by a power-law extrapolation. Furthermore, it provides good agreement between the scattering curves obtained by both techniques in the overlapping *q* interval. This is especially important for complex scattering patterns, where power-law trends for high-*q*_*x*_ USANS are not so obvious.

## Figures and Tables

**Table 1:** Small-angle neutron scattering instruments used

	Yellow Submarine	MAUD
Neutron source	BRR†	LVR-15‡
Type	SANS	USANS
Collimation	Pinhole	Slit
Sample-to-detector distance (m)	1.3 and 5.4	0.9
Wavelength (nm)	0.3–1.2	0.209
Monochromatization (%)	12–30	2–10
*q* range (nm^−1^)	0.05–4	0.002–0.2
Flux (s^−1^ cm^−2^)	5 × 10^7^	5 × 10^4^
Beam section (mm^2^)	40 × 40	5 × 40
Detector type	2D-PSD	1D-PSD
Detector size (cm^2^)	64 × 64	5 × 17

† Budapest Research Reactor.

‡ Research reactor at Řež near Prague.
